# Conformational folding and disulfide bonding drive distinct stages of protein structure formation

**DOI:** 10.1038/s41598-018-20014-y

**Published:** 2018-01-24

**Authors:** Jian-Min Lv, Shou-Qin Lü, Zu-Pei Liu, Juan Zhang, Bo-Xuan Gao, Zhen-Yu Yao, Yue-Xin Wu, Lawrence A. Potempa, Shang-Rong Ji, Mian Long, Yi Wu

**Affiliations:** 1 MOE Key Laboratory of Cell Activities and Stress Adaptations, School of Life Sciences, Lanzhou University, Lanzhou, 730000 P.R. China; 2 Center for Biomechanics and Bioengineering, Key Laboratory of Microgravity (National Microgravity Laboratory), and Beijing Key Laboratory of Engineered Construction and Mechanobiology, Institute of Mechanics, Chinese Academy of Sciences, Beijing, 100190 P.R. China; 3 Roosevelt University College of Pharmacy, Schaumburg, IL 60173 USA; 4 MOE Key Laboratory of Environment and Genes Related to Diseases, School of Basic Medical Sciences, Xi’an Jiaotong University, Xi’an, Shaanxi 710061 P.R. China; 5 Key Laboratory of Preclinical Study for New Drugs of Gansu Province, Lanzhou University, Lanzhou, 730000 P.R. China

## Abstract

The causal relationship between conformational folding and disulfide bonding in protein oxidative folding remains incompletely defined. Here we show a stage-dependent interplay between the two events in oxidative folding of C-reactive protein (CRP) in live cells. CRP is composed of five identical subunits, which first fold spontaneously to a near-native core with a correctly positioned C-terminal helix. This process drives the formation of the intra-subunit disulfide bond between Cys36 and Cys97. The second stage of subunit folding, however, is a non-spontaneous process with extensive restructuring driven instead by the intra-subunit disulfide bond and guided by calcium binding-mediated anchoring. With the folded subunits, pentamer assembly ensues. Our results argue that folding spontaneity is the major determinant that dictates which event acts as the driver. The stepwise folding pathway of CRP further suggests that one major route might be selected out of the many in theory for efficient folding in the cellular environment.

## Introduction

Many secretory proteins possess disulfide bonds. A key issue in understanding their native structure formation, *i.e*. oxidative protein folding, is the causal relationship between conformational folding and disulfide bonding^1^. Though controversial^2^, recent *in vitro* experimental^3^ and computational studies^4^ conclude that conformational folding drives disulfide bonding. However, it is not clear how general this conclusion is and whether this conclusion can be extrapolated to oxidative folding *in vivo*, which differs from that *in vitro* in several important ways^5^.

C-reactive protein (CRP) is a marker of inflammation with roles in host defense^6,7^. In response to inflammatory stimuli, the blood level of CRP can rise up to 1000-fold to over 500 μg/ml^6^, accounting for ~4 % of proteins secreted by hepatocytes^8^. CRP is assembled non-covalently by five identical subunits^9^. Each subunit folds into a single globular domain of 23 kD containing only two cysteines that form an intra-subunit disulfide bond^9^. Moreover, CRP is non-glycosylated and, using recombinant technologies, can be secreted by *E. coli* cells in the native conformation^10^. These suggest that the folding of CRP is highly efficient and probably self-sufficient. Attempts to refold native CRP from urea-denatured subunits *in vitro* have failed^11–13^, however, implying there is a mandatory need for cellular factors to fold CRP into its native structure.

In this study, we have examined the oxidative folding and assembly of CRP subunits in COS-7 and *E. coli* cells. In the spontaneous stage of CRP folding, conformational folding drives the formation of the intra-subunit disulfide bond. The subsequent, non-spontaneous folding stage ensues only after and depending upon the proper formation of the intra-subunit disulfide bond to accomplish the large conformational remodeling. Our findings suggest that disulfide bonding driven by conformational folding may be a general feature in case of spontaneous structure formation, while the converse could hold true in scenario where major structural rearrangement is required.

## Results

### Subunit folding and pentamer assembly occur sequentially

**Figure 1:**
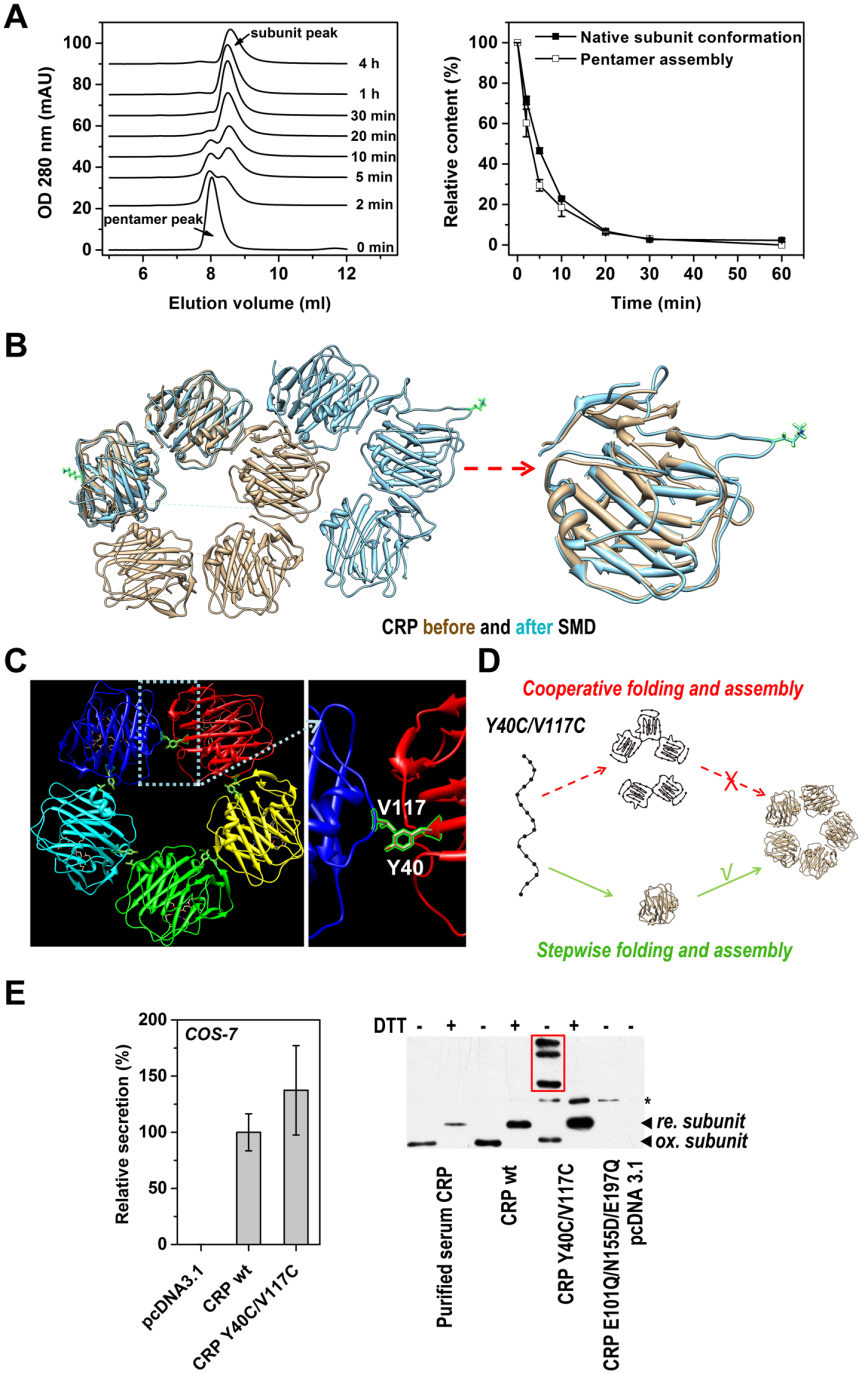
Subunit folding precedes pentamer assembly. (**A**) CRP were diluted to 100 μg/ml in buffers containing 8 M urea, 5 mM EDTA and sampled at the indicated times. The assembly states of the samples were analyzed by size-exclusion chromatography with a Superdex G75 column (left panel). To stabilize the native pentameric state, samples were eluted with 8 M urea containing 2 mM Ca, which prevents CRP from urea-induced denaturation^13^. Moreover, dissociated subunits taken at any time point are unable to regenerate the native pentamer under such conditions^11^. The content of native subunit conformation was determined with specific ELISA using 1D6 mAb and compared with pentamer content (right panel). ELISA samples were taken from the same reactions used in SEC and diluted in calcium containing buffer^13^. Previous study^11^ and our refolding attempts consistently demonstrate that the denatured subunits are unable to refold *in vitro* to any significant extent. At the earlier time points, the content of native subunit conformation was higher than that of pentameric assembly, suggesting that pentamer dissociation occurred before subunit unfolding. (**B**) The pentameric assembly (left panel) and subunit structure (right panel) of CRP before (pale gold) and after (light blue) constant velocity (0.01 Å/ps) steered molecular dynamics (SMD) simulation by NAMD 2.9^31^ using a Charmm27 all-atom force field^32^. The structure was solvated by TIP3P water with a buffer distance of 25 Å, leading to a model system of ~280,000 atoms. Force (300 pN/Å with a spring constant K of 4.35 kcal∙(mol Å^2^)^−1^) was applied through the two highlighted Lys23 on opposite subunits in the pentamer. At the end of 65-ns simulation, the pentameric assembly was disrupted while the native subunit structure was largely preserved. (**C**) Y40 and V117 are juxtaposed at the assembly interface between two adjacent subunits in the crystal structure of CRP (PDB 1B09). They were mutated to cysteines to introduce engineered inter-subunit disulfide bonds that are compatible with the pentameric structure. (**D**) A schematic diagram describing that efficient secretion of CRP Y40C/V117C only occurs when pentameric CRP is assembled by (near) folded subunits. (**E**) Efficient secretion of CRP Y40C/V117C mutant by COS-7 cells was detected by ELISA (left panel) and by immunoblotting (right panel). The formation of the inter-subunit disulfide bond in CRP Y40C/V117C was revealed by the presence of oligomer bands sensitive to reduction (marked by the red box) in immunoblotting of the conditioned media using clone8 mAb. * indicates non-specific signal. Each data point represents the mean of data obtained from at least 3 independent experiments and is presented as mean +/− S.E.

We first clarified whether subunit folding and pentamer assembly of CRP proceed sequentially or cooperatively. The process of native structure formation could be mirrored at least partially by the reverse process, *i.e*. denaturation. We thus induced denaturation of CRP experimentally using urea (Fig. 1A) or computationally using simulated forces (Figs 1B and S1). Both approaches suggested that pentamer dissociation precedes subunit unfolding, leading us to hypothesize that the native structure formation of CRP also proceeds sequentially, with CRP subunits folding first, followed by assembly into the pentamer.

To test the above hypothesis in live cells, we designed two secretory CRP mutants to contain engineered inter-subunit disulfide bonds, carefully positioned so not to disturb essential inter-subunit salt bridge interactions^9^ (Figs 1C and S2). We rationalized that if subunit folding and assembly of CRP occur cooperatively, covalent disulfide bonding between partially folded subunits would generate longer and branched polypeptides, strongly hindering their subsequent folding and assembly cycles. If this occurred, because of the tight quality control in eukaryotic cells^5^, there would be significantly lower yield of native CRP secreted into conditioned media. In contrast, if CRP pentamer assembles only after nearly folded subunits form, the formation of the engineered inter-subunit disulfides would be compatible with the native pentameric structure and would be secreted efficiently (Fig. 1D). Our results revealed secretion of both mutants by COS-7 cells were comparable to that of wild-type CRP (CRP wt), and their inter-subunit disulfide bonds were formed as expected (Figs 1E and S2). Of note, disruption of residues involved in inter-subunit salt bridge interactions mediating pentamer assembly^9^, completely prevented CRP secretion (Fig. 1E). These data are consistent with the conclusion that native CRP structure is formed sequentially, with folding of CRP subunits preceding pentamer assembly.

### Folding of strands C to H drives the intra-subunit disulfide bonding

**Figure 2:**
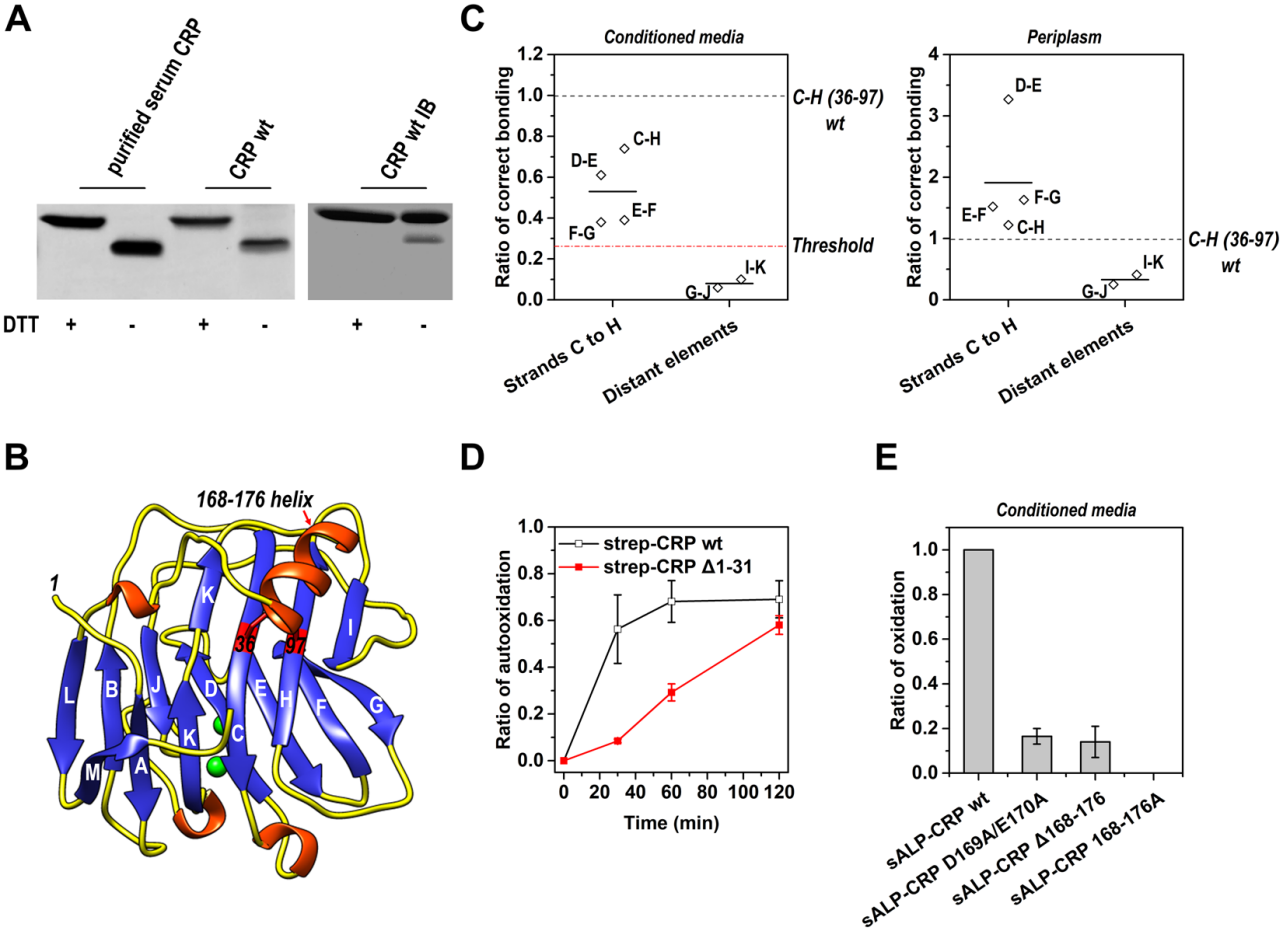
Folding of strands C to H drives the formation of the intra-subunit disulfide bond. (**A**) Inclusion bodies (IB) formed by CRP wt in *E. coli* cytoplasm were separated and solubilized by 8 M urea. After passing through a desalting column to remove urea, the proteins were incubated in 20 mM Tris, 140 mM NaCl, pH 7.4 at 4 °C overnight (left panel). Alternatively, IB without urea solubilization were incubated overnight (right panel). Samples with or without DTT reduction were analyzed by non-reducing SDS-PAGE and immunoblotting with 3H12 mAb. Oxidized subunits migrated faster than their reduced counterparts^14,28^. Urea-solubilized CRP subunits in IB were in reduced state, but were oxidized spontaneously following urea removal. (**B**) Crystal structure of CRP subunit (PDB 1B09). The hydrophobic core of the subunit is composed of a two-layered β sheet comprising strands A to K. Cys36 and Cys97 are located on strands C and H, respectively. They form an intra-subunit disulfide bond that is covered by a.a. 168–176 helix. (**C**) Secretory CRP mutants with Cys36 and Cys97 mutated to serines and disulfide bonds introduced between the indicated strands were expressed in *E. coli* cells (Table S1). Two strands were considered to be in close proximity only when the introduced disulfide bond formed more efficiently than that of CRP wt (in periplasm; left panel) or the threshold value (in conditioned media; right panel). The threshold value (indicated by the red dotted line) was assigned as the averaged ratio of disulfide bonding between distant elements plus 3 times S.D. to account for the stochastic oxidation. Strands C to H appeared to be in near native configuration before Cys36-Cys97 bonding. (**D**) The spontaneous formation of the intra-subunit disulfide bond in purified CRP wt and Δ1-31 mutant with strep tags. The absence of 1–31 (strands A and B) drastically reduced the speed of Cys36-Cys97 bonding. (**E**) The intra-subunit disulfide bond formation in CRP wt and 168–176 helix mutants secreted into conditioned media by *E. coli* cells. Mutating D169 and E170 (D169A/E170A), deleting a.a. 168–176 (Δ168-176), or replacing a.a. 168–176 with alanines (168–176 A) all dramatically impaired the intra-subunit disulfide bonding. Data were presented as mean +/− S.E. Each data point represents the mean of data obtained from at least 3 independent experiments.

Each CRP subunit contains two cysteines, *i.e*. Cys36 and Cys97, that form an intra-subunit disulfide bond. To explore the folding status prior to the formation of the intra-subunit disulfide bond, we expressed non-secretory CRP in the cytoplasm of *E. coli* cells. Cytoplasmic CRP subunits were in reduced state and formed inclusion bodies. The inclusion bodies were isolated and solubilized using 8 M urea. Though urea-solubilized CRP subunits were still in reduced state, their intra-subunit disulfide bonds were spontaneously recovered when urea was removed by desalting columns (Fig. 2A). Even though intra-subunit disulfide bonds did properly from in these CRP subunits, native pentameric structure could never be regenerated, being consistent with previous reports^11,14^. Since the disulfide bond-forming cysteines are far apart in primary sequence, their spontaneous juxtaposition for correct and efficient bonding suggests that CRP subunits have the intrinsic capacity to reach a state of long-range, yet partially native conformation. It is possible that the denatured CRP subunit can spontaneously fold to this state that drives disulfide bonding, consistent with the folded-precursor mechanism for disulfide-containing proteins as supported by recent experimental^3^ and computational investigations^4^. Alternatively, the quasi-stochastic mechanism of disulfide bonding may conversely drive establishment of this state by biasing folding pathways^2^.

To distinguish the two mechanisms, we designed three sets of experiments. First, cytoplasmic inclusion bodies were isolated without urea solubilization, and the redox status of CRP subunits were examined. Spontaneous recovery of the intra-subunit disulfide bond could still be observed over time albeit less efficiently (Fig. 2A). This may be due to refolding of the subunits in inclusion bodies, or more likely because the subunits were already in a conformation state compatible with Cys36-Cys97 bonding before leaving the reductive cytoplasm. This favors the folded-precursor mechanisms. In the crystal structure of CRP, Cys36 and Cys97 are held in place by β-strands C and H, as part of the jellyroll-like core formed by a two-layered β sheet that includes strands A to K (Fig. 2B). Therefore, the folded-precursor mechanism would predict that the core encompassing strands C to H is folded prior to Cys36-Cys97 bonding, whereas the quasi-stochastic mechanism would predict the converse.

To determine the folding state of strands C to H before Cys36-Cys97 bonding in live cells, disulfide scanning experiments^15–17^ were next performed. For this analysis, CRP mutants were made by replacing Cys36 and Cys97 with serine residues and by introducing a new disulfide bond to form between juxtaposed residues on selected strands (Fig. 2B; Table S1). These mutants were fused with a signal peptide to allow their secretion through the oxidative periplasm of *E. coli* cells so to allow formation of disulfide bonds. Disulfide bonds introduced between all neighboring pairs among strands C to H were found to be formed with high efficiency (Figs 2C and S3). By contrast, those introduced between distant structural elements could be barely formed. These results suggest that strands C to H are able to fold to a near native state that is compatible with but independent of Cys36-Cys97 bonding.

In the third set of experiments, a panel of truncation mutants were prepared and tested for their cytoplasmic stability and for spontaneous recovery of intra-subunit disulfide bonds. Truncation of strands A and B markedly reduced the cytoplasmic stability (Figure S4) and slowed the rate of disulfide bond formation in purified mutants (Fig. 2D). These data suggest that strands A and B can accelerate but are nevertheless dispensable for the folding of strands C to H. By contrast, truncation of the entire C-terminal half (*i.e*. a.a. 105–206) did not affect cytoplasmic stability (Figure S4), highlighting the self-sufficiency of strands C to H for correct folding. Despite this finding, the C-terminal sequence of a.a. 165–179 was unexpectedly found to be essential for the formation of the intra-subunit disulfide bond (Figure S5). This sequence contains a motif (a.a. 168–176) that folds as an α-helix over Cys36 and Cys97 (Fig. 2B), providing the structural basis for its modulation on disulfide bonding. Accordingly, full-length mutants with targeted alterations in a.a. 168–176 helix also exhibited severely impaired formation of the intra-subunit disulfide bond in *E. coli* cells (Fig. 2E). Together, we conclude that Cys36 and Cys97 bonding is driven by folding of the N-terminal strands C to H and correct positioning of the C-terminal helix.

### The intra-subunit disulfide bonding licenses integration of strands J and K

**Figure 3:**
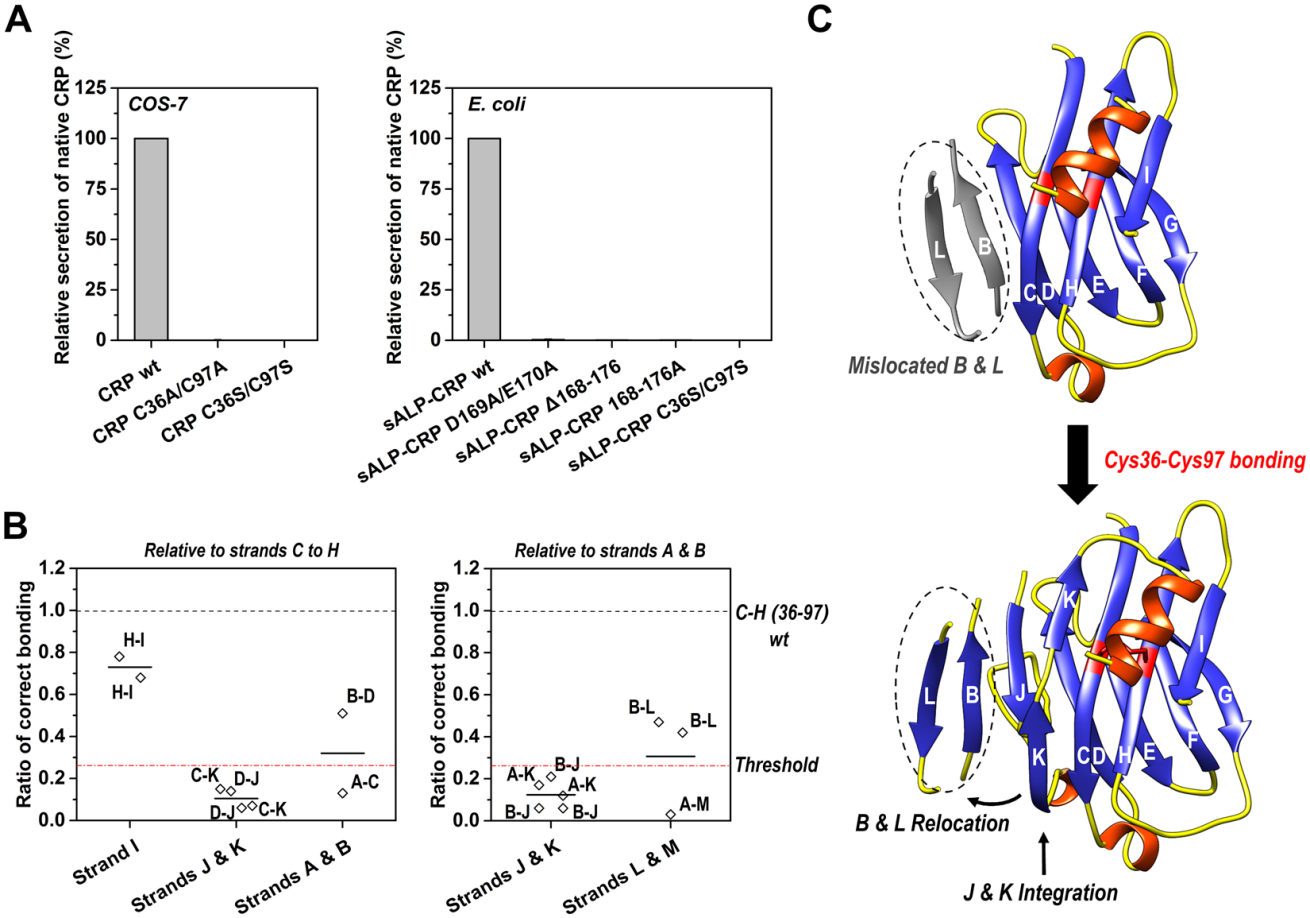
The formation of the intra-subunit disulfide bond licenses the subsequent core rearrangement. (**A**) Secretory CRP mutants with N-terminal signal peptide of CRP or ALP (Alkaline phosphatase) were expressed by COS-7 (left) or *E. coli* cells (right). Mutating cysteines or a.a. 166–178 helix prevented the secretion of native CRP. (**B**) The configuration of strands in addition to C to H before Cys36-Cys97 bonding was examined with appropriate CRP mutants (Table S2). Beside strand I, other strands were found to be either misassembled or unfolded. (**C**) A schematic diagram summarizes the core configuration before and after formation of the intra-subunit disulfide bond. Data were presented as mean +/− S.E. Each data point represents the mean of data obtained from at least 3 independent experiments.

Though the formation of the intra-subunit disulfide bond appears to be the result of conformational folding, mutating Cys36 and Cys97 to alanines or serines nevertheless abrogated the secretion of native CRP by both COS-7 and *E. coli* cells (Fig. 3A). Mutating the 168–176 helix also prevented the secretion of native CRP. The expression levels of those mutants, however, were not different from that of CRP wt. These results indicate that certain events at later stages of subunit folding depend on the intact intra-subunit disulfide bond. We reasoned that such events would be related to structural elements that are not properly folded until the Cys36-Cys97 bond forms.

Disulfide scanning was thus conducted to determine the folding status of other strands in the core before Cys36-Cys97 bonding (Figs 3B and S6; Table S2). Strand I, in addition to strands C to H (Fig. 2C), was found to be correctly assembled. However, strands J and K, located at the middle of the core, were not in place. These two strands are key to the integrity of the core by mediating the assembly of the N-(A, B) and C-terminal strands (L, M), with strands C to I (Fig. 2B). The relative positioning of strands A and M could not be detected, suggesting they were similarly out of position. By contrast, the N-terminal strand B appeared to directly contact with the C-terminal strand D, bypassing the mediation of strand J, and showed a near-native proximity to the C-terminal strand L. Taken together, these results depict a scenario in which the subunit core begins forming by correctly assembling strands C to I with the rest strands misassembled or unfolded before Cys36-Cys97 bonding. Consequently, a large conformational remodeling is required for the subsequent integration of strands J and K into their native position with the relocation of strands B and L as the prerequisite (Fig. 3C). As such, the formation of a covalent disulfide bond at an optimized position can be mandatory to keep the right configuration of strands C to I during that remodeling.

To model how the integration of strands J and K might perturb core strands C to I and the impact of the intra-subunit disulfide bond, we performed steered molecular dynamics simulation using the structure of CRP subunit with or without Cys36-Cys97 bonding (Figs S7 and S8). The pulling force was applied to the sequence flanking strand J with N-terminus as the fixed point. Strands J and K were disintegrated almost simultaneously by the force. Interestingly, strand C was also taken away from the core together with strand K likely due to their strong interaction (Figure S9). These would suggest that the integration of strands J and K may disrupt the correct configuration of the core. With the presence of Cys36-Cys97 bonding, however, the stability of the core is markedly enhanced as evidenced by the much longer time required for the force-induced disassembly of strand C versus that without (Figure S8). The enhanced stability could be explained by the covalent linking that synchronizes the motion of strand C and the rest of the core, rendering a stronger resistance to perturbation. Indeed, upon the disassembly of strand C, the remaining core strands were also disassembled when the disulfide bond was present; while they were not affected when the disulfide bond was absent.

### Calcium binding guides the integration of strands J and K

**Figure 4:**
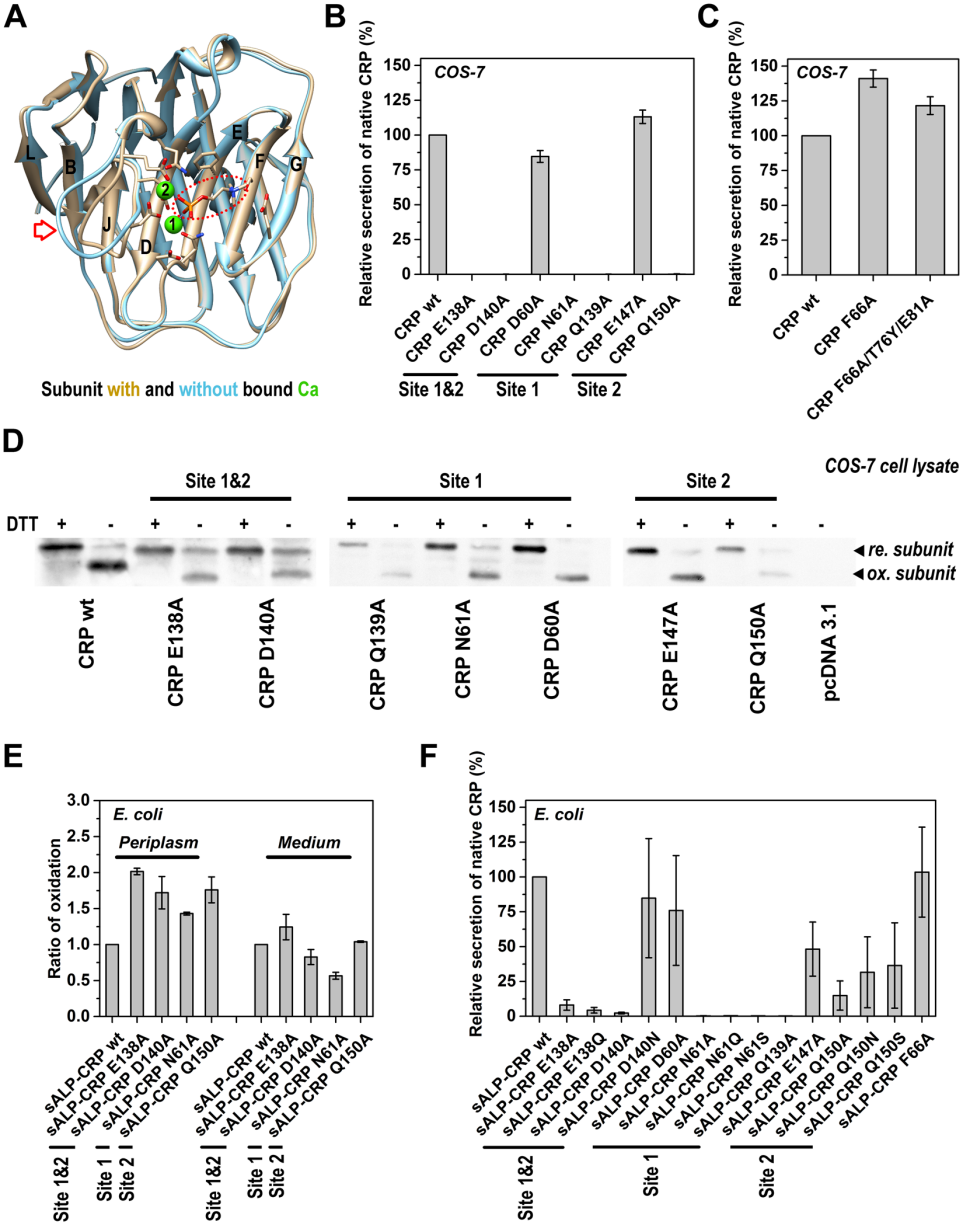
Calcium binding finalizes subunit folding. (**A**) A schematic picture superimposing structures of CRP subunit with (PDB 1B09) and without bound calcium (PDB 1LJ7). The red arrow indicates the region where major structural differences were noted. Red circles identify bound PC ligand. Green spheres represent bound calcium ions. Secretory CRP mutants with mutations at the calcium binding residues (**B**) or PC binding residues (**C**) were expressed by COS-7 cells. D60A and E147A mutations did not impair calcium binding^19^ and secretion of native protein. All other calcium binding site mutants were not secreted. PC binding site mutants, by contrast, were secreted normally. Mutating calcium binding sites showed little effect on the formation of intra-subunit disulfide bond in COS-7 (**D**) and *E. coli* cells (**E**). (**F**) Secretory CRP mutants with mutations in the calcium binding sites were expressed by *E. coli* cells. Most mutants that could not be secreted by COS-7 cells were detected in conditioned media of *E. coli* cells in the native conformation. Each data point represents the mean of data obtained from at least 3 independent experiments and is presented as mean +/− S.E.

The structure and activities of CRP are critically dependent on calcium^6,9,18^. The presence of calcium can even prevent the denaturation of CRP induced by 8 M urea^13^. Moreover, CRP binds in a calcium-dependent manner to most of its ligands, including phosphocholine (PC). To address whether calcium plays any role in cellular folding of CRP, a series of mutants with point mutations that disrupt either or both calcium binding sites were constructed (Fig. 4A). Except for mutants D60A and E147A, (both of which have been reported not to affect calcium binding^19^), none of the other mutants tested were secreted by COS-7 cells (Fig. 4B). While PC-binding and calcium-binding sites are closely associated in the CRP structure^20^, disruption of residues influencing PC-binding did not impair CRP secretion (Fig. 4C). These data indicate that calcium binding is critical for folding of native CRP.

We next asked whether calcium affected the early (spontaneous) or the latter (non-spontaneous) stages of CRP folding. We observed that intra-subunit disulfide bonds were normally formed by calcium-binding site mutants in both COS-7 and *E. coli* cells (Figs 4D, E and S10). Moreover, the spontaneous formation of the intra-subunit disulfide bond *in vitro* did not require calcium (Fig. 2A). These indicate that calcium has little if any effect on spontaneous subunit folding before Cys36-Cys97 bonding.

Following Cys36-Cys97 bonding, the major folding event is the integration of strands J and K into the core. The success of this event, however, depends on a correct topology between the structural elements involved. The establishment of such a topology likely also represents the rate-limiting step in CRP folding. Interestingly, while most residues constituting calcium-binding sites are located in the loop connecting strands J and K, two residues are located in strand E of the core. We thus hypothesized that calcium binding might guide the integration event by anchoring strands J and K to the right position relative to the core. As such, this hypothesis would predict that (i) a defect in calcium binding-mediated anchoring might be partly rescued by loosening quality control to prolong the topology searching and folding cycles before termination^21^; and (ii) the calcium binding site composed of residues Q139, N61 and D60 (*i. e*. calcium binding site 1) is more critical since only this site involves residues in the core. The quality control system in prokaryotic *E. coli* cells is less strict than that of eukaryotic COS-7 cells. As predicted, in contrast to COS-7 cells, *E. coli* cells could secret most calcium-binding site mutants in the native conformation albeit with reduced efficiency (Fig. 4F). The folding defect caused by mutations at key residues exclusively involved calcium binding site 1, however, was barely rescued. These results together suggest that calcium binding accelerate the establishment of an integration-priming configuration.

## Discussion

**Figure 5:**
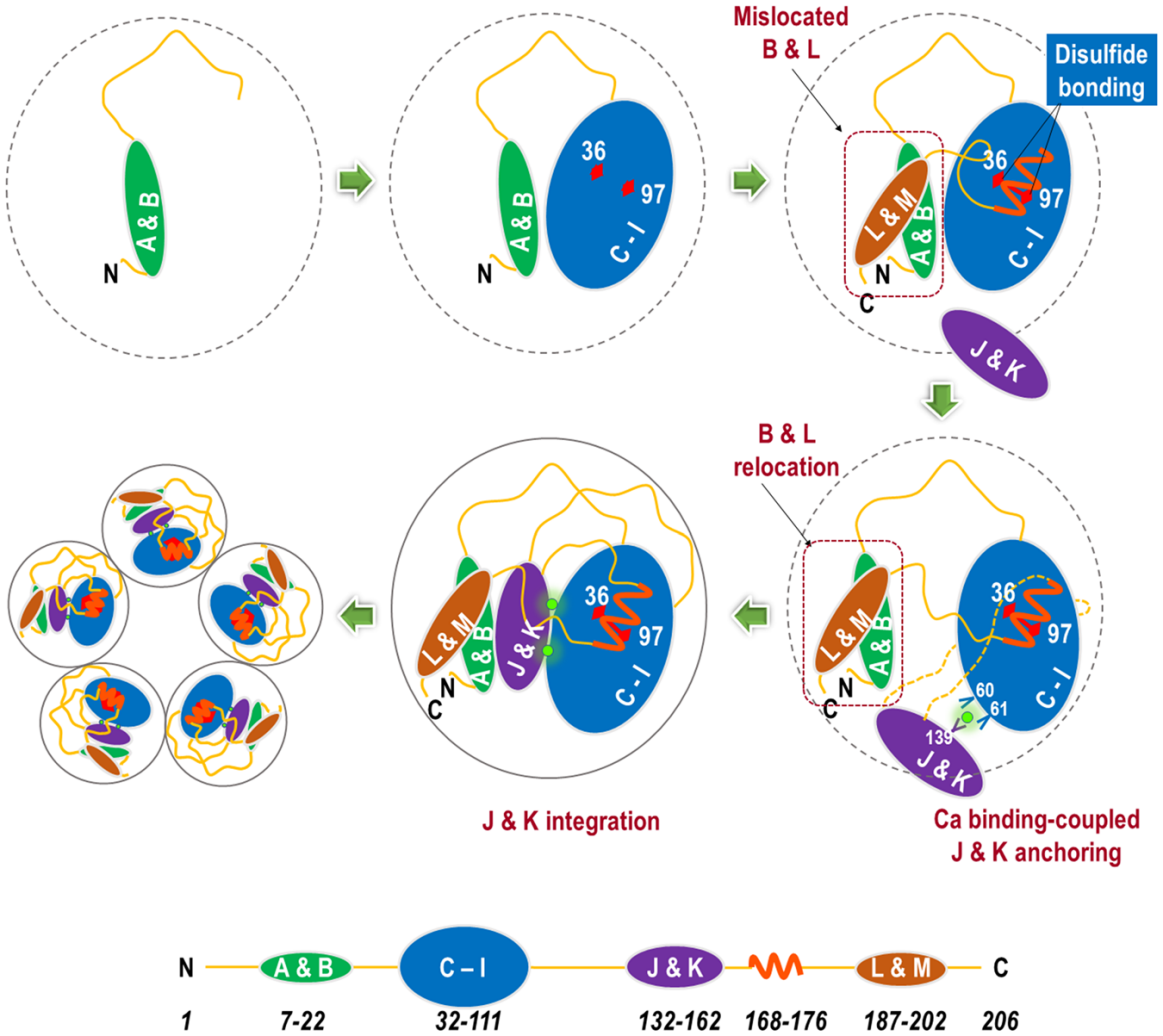
Schematic illustration of the folding pathway of CRP. The initial folding of strands A and B promotes correct assemblage of strands C to I. This brings Cys36 and Cys97 in close proximity and their bonding is ensued with the coverage by a.a. 168–176 helix. At this stage, strands B and L are misassembled in relation to strands C to I. Subsequently, calcium binding by Asp60, Asn61 on strand E and Gln139 on strand J guides the assemblage of strands J and K with strands C to I, displacing strands B and L to the right location. After the integration of strands J and K, CRP subunit attain a near-native conformation that allows pentamer assembly.

Our results reveal that the cellular production of pentameric CRP proceeds in a stepwise manner (Fig. 5). We show that individual CRP subunits must first fold to a near-native structure before they can be assembled to produce the pentamer (Fig. 1). This is not so unexpected as the inter-subunit assembly mainly involves peripheral motifs, including a.a. 115–123 loop of one subunit, and a.a. 40–42 loop and a.a. 197–202 strand of another subunit^20^. The correct interactions among these distantly located motifs is *per se* an indication for assembly by near-folded subunits. Moreover, the modest interfacial area of less than 900 Å^2^ suggests an inherent capacity of CRP subunits to rotate within the pentamer with subtle conformational rearrangements^20^. All these data support the notion that the structural integrity of a CRP subunit can be independent, to a certain degree, of pentameric assembly.

The folding of CRP subunit appears to be a two-stage process in live cells (Figs 2 and 3). In the first stage, spontaneous conformational folding of the core comprising strands C to I and the C-terminal helix drives the formation of the intra-subunit disulfide bond. However, the second stage of subunit folding is instead dependent on this disulfide bond, which helps in keeping the correct core configuration for the integration of strands J and K (Fig. 3). There are controversies on whether conformational folding drives disulfide bonding (*i.e*. folded-precursor mechanism)^3,4^ or vice versa (*i.e*. quasi-stochastic mechanism)^2^. Theoretically, folded-precursor mechanism exploits structural information intrinsically encoded by primary sequence, and would therefore be more plausible and efficient than quasi-stochastic mechanism in dictating disulfide bond formation. This might hold true in particular for cysteines located far apart in primary sequence, as exemplified here by the first spontaneous folding stage of CRP subunit and also by the framework model depicting oxidative folding of BPTI^22^. Once formed, the intra-subunit disulfide bond of CRP subunit licenses the large conformational remodeling that underlies the success of the second, non-spontaneous folding stage. Such a manifestation differs from quasi-stochastic mechanism, but similarly highlights the importance of disulfide bond formation in directing conformational folding with extensive restructuring. In this regard, it is worth noting that most, if not all model substrates examined so far in oxidative protein folding are able to refold spontaneously *in vitro*^1–4,22^. The lack of a strong folding barrier to overcome would obscure the role of disulfide bonding, and might represent a major hurdle in clarifying their causal relationship.

In summary, our results reveal an intimate interplay between conformational folding and disulfide bonding with each acting as the driver in a stage-dependent manner. While disulfide bonding is the result of conformational folding in spontaneous phase, our data suggests that formation of these covalent bonds could drive the non-spontaneous phase that requires large conformation remodeling even in the presence of full set of cellular factors that assists folding. Therefore, only with appropriate identification of folding intermediates and characterization of the native state, can we depict a complete picture of the disulfide-coupled protein folding. Moreover, the folding process of CRP demonstrated herein is consistent with the foldon model^23^, in which protein folds through discrete intermediates. Despite numerous folding routes in theory, proteins may be co-evolved with and shaped by the quality control system to fold through specific pathways for efficient cellular production.

## Methods

### Reagents

Human native CRP (CRP, purity > 99 %) purified from ascites were purchased from the BindingSite (Birmingham, UK). Denaturation of CRP was performed by urea-chelation treatment^24^. Anti-CRP mAbs 1D6, 8D8 and 3H12 were prepared as described^25^. 1D6 and 8D8 recognize conformational epitope in native CRP, whereas 3H12 binds to a linear epitope exposed only in dissociated subunits^25^. Other reagents were from Sigma-Aldrich (St. Louis, MO) unless otherwise stated.

### Expression of CRP mutants

For expression in *E. coli* (BL21) cells, coding sequences of wild-type and mutant CRP were cloned into pET42c plasmids. Where necessary, the N-terminus of CRP was fused with signal peptide of alkaline phosphatase (ALP) to enable secretion. CRP overexpressed BL21 cells were induced with 0.5 mM IPTG for 24 h at 16 °C. Cells were centrifugated at 5000 g for 5 min, and supernatants were collected as conditioned media. Periplasm of pelleted cells was isolated as described^26^.

For expression in COS-7 cells, coding sequences of wild-type and mutant CRP were cloned into pcDNA3.1 plasmids (Invitrogen). COS-7 cells were transfected with CRP expression plasmids by Lipofectamine 2000 (Invitrogen) and cultured for 48 h before experimentation.

### Quantification of secreted CRP

The levels of CRP secreted in conditioned media of *E. coli* or COS-7 cells were determined with sandwich ELISA using conformational-specific mAbs^27^. Briefly, microtiter wells were coated with 5 μg/ml sheep anti-human CRP polyclonal antibody (BindingSite) overnight at 4 °C. All the following incubation steps were conducted at 37 °C. After blocking the wells with 1 % BSA, conditioned media diluted in blocking buffer were added for 1 h. Captured CRP were detected with appropriate mAb and a HRP-conjugated secondary antibody. Wells were developed with TMB and stopped with 1 M H_2_SO_4_.

### Analysis of redox states of CRP subunits

Each CRP subunit has only two cysteine residues (Cys36 and Cys97) that form an intra-subunit disulfide bond. The redox states of CRP subunits (*i.e*. whether the intra-subunit disulfide bond forms) were determined by 12 % non-reducing SDS-PAGE^14^, in which the reduced subunit ran slower than the non-reduced counterpart^14,28^. To freeze the original redox state, 5 mM N-ethylmaleimide (NEM) was added upon cell lysis or sampling of conditioned media to protect free cysteines from oxidation. For experiments using inclusion bodies, they were isolated by centrifugation in the absence of NEM, then solubilized with 8 M urea, and finally passed through the desalting columns to remove the denaturant. After treatment with or without 10 mM DTT for 1 h, samples were subjected to 12 % non-reducing SDS-PAGE and bands were visualized by coomassie blue staining or immunoblotting.

### Steered molecular dynamics (SMD) simulations

Crystal structures of CRP pentamer with (PDB code: 3PVO^29^) or without calcium ion (PDB code: 1LJ7^30^) were employed for the MD simulations. Two sets of simulation systems were built as shown in Table S3, one for pentamer (penta) systems and the other for monomer (mono) systems. The calcium ions were deleted directly from crystalized 3PVO structures for building a penta- or mono-3PVO-(C_a_^2+^)^−^ system. Chain A of 3PVO was adopted as CRP monomer for the two monomer systems, and the mutations of both Cys36 and Cys97 to Ser were performed for disrupting the disulfide bond of Cys36-Cys97 in the mono-3PVO-(S-S)^−^ system. Each simulation system was built up by solvating the target molecule(s) in a rectangular water box and neutralized with ~100 μM C_a_Cl^2+^. The NAMD^31^ program with CHARMM27^32^ all-atom force field was used for the simulations.

An integration time step of 2 femtosecond (*fs*) and the periodic boundary conditions were applied in the equilibration simulations. A smooth (10–12 Å) cutoff and the Particle Mesh Ewald (PME) method were employed to calculate van der Waals forces and full electrostatics, respectively. A 310 K heat bath was manipulated using a Langevin thermostat, and 1 atm pressure was controlled by the Nose-Hoover Langevin piston method. Prior to the equilibration process, energy minimization with 50,000 steps of fixed backbone atoms was followed by an additional 100,000 steps with all atoms free, and the system was heated from 0 to 310 K by 31 K increments every 2 picoseconds (*ps*). Equilibration of 100 nanosecond (*ns*) was performed for each system. Then, steered MD (SMD) simulations were conducted based on the final state of respective 100-*ns* equilibration. For pentamer systems, a constant force of 600 piconewton (*pN*) was applied on the C_α_ atom center of Glu138-Asp140 peptide of one subunit and the N-terminal Cln1-C_α_ atoms of other four subunits were fixed, and the force direction was along the vector from the geometry center of fixed atoms to the pulled atoms. For monomer systems, a constant force of 400 *pN* was applied on the C_α_ atom center of Glu138-Asp140 peptide with its N-terminal Gln1-C_α_ atom fixed under the same force direction definition as that for pentamer system.

Relative and absolute root mean squared deviation (RMSD) of the pulled subunit were used to quantify its dissociation from the pentamer and its own structural disruption, respectively, during the SMD simulations of pentamer systems. The former and the latter were calculated upon the backbone alignment of the respective four fixed subunits and the pulled subunit itself. The extension, defined as the distance between the geometric centers of fixed and pulled atoms, was calculated for evaluating the structural unfolding during the SMD simulations of monomer systems. In addition, the interaction strength between adjacent strands were evaluated by the numbers of hydrogen bond (Hbond) with a donor-acceptor distance < 3.0 Å and a donor-hydrogen-acceptor angle < 45°. All system construction, residue site mutations, structural analyses and visualization were performed using the VMD program^33^.

### Data availability statement

All the data of manuscript is available.

## Electronic supplementary material

Supplemental data

## References

[1] Narayan M (2012). Disulfide bonds: protein folding and subcellular protein trafficking. FEBS J.

[2] Welker E, Wedemeyer WJ, Narayan M, Scheraga HA (2001). Coupling of conformational folding and disulfide-bond reactions in oxidative folding of proteins. Biochemistry.

[3] Kosuri P (2012). Protein folding drives disulfide formation. Cell.

[4] Qin M, Wang W, Thirumalai D (2015). Protein folding guides disulfide bond formation. Proc Natl Acad Sci USA.

[5] Balchin D, Hayer-Hartl M, Hartl FU (2016). *In vivo* aspects of protein folding and quality control. Science.

[6] Pepys MB, Hirschfield GM (2003). C-reactive protein: a critical update. J Clin Invest.

[7] Bottazzi B, Doni A, Garlanda C, Mantovani A (2010). An integrated view of humoral innate immunity: pentraxins as a paradigm. Annu Rev Immunol.

[8] MacIntyre SS, Schultz D, Kushner I (1983). Synthesis and secretion of C-reactive protein by rabbit primary hepatocyte cultures. Biochem J.

[9] Volanakis JE (2001). Human C-reactive protein: expression, structure, and function. Mol Immunol.

[10] Tanaka, T., Horio, T. & Matuo, Y. Secretory production of recombinant human C-reactive protein in Escherichia coli, capable of binding with phosphorylcholine, and its characterization. *Biochem Biophys Res Commun* **29**5, 163–166, S0006-291X(02)00622-8[pii] (2002). 10.1016/s0006-291x(02)00622-8 12083784

[11] Kresl, J. J., Potempa, L. A. & Anderson, B. E. Conversion of native oligomeric to a modified monomeric form of human C-reactive protein. *Int J Biochem Cell Biol* **3**0, 1415–1426, S1357272598000788[pii] (1998). 10.1016/s1357-2725(98)00078-8 9924810

[12] Potempa LA, Siegel JN, Fiedel BA, Potempa RT, Gewurz H (1987). Expression, detection and assay of a neoantigen (Neo-CRP) associated with a free, human C-reactive protein subunit. Mol Immunol.

[13] Potempa LA, Maldonado BA, Laurent P, Zemel ES, Gewurz H (1983). Antigenic, electrophoretic and binding alterations of human C-reactive protein modified selectively in the absence of calcium. Mol Immunol.

[14] Wang MY (2011). A redox switch in C-reactive protein modulates activation of endothelial cells. FASEB J.

[15] Sato TK, Tweten RK, Johnson AE (2013). Disulfide-bond scanning reveals assembly state and beta-strand tilt angle of the PFO beta-barrel. Nat Chem Biol.

[16] Ramachandran R, Tweten RK, Johnson AE (2004). Membrane-dependent conformational changes initiate cholesterol-dependent cytolysin oligomerization and intersubunit beta-strand alignment. Nat Struct Mol Biol.

[17] Greene NP (2007). Cysteine scanning mutagenesis and disulfide mapping studies of the TatA component of the bacterial twin arginine translocase. J Biol Chem.

[18] Ma X, Ji SR, Wu Y (2013). Regulated conformation changes in C-reactive protein orchestrate its role in atherogenesis. Chinese Sci Bull.

[19] Suresh MV, Singh SK, Agrawal A (2004). Interaction of calcium-bound C-reactive protein with fibronectin is controlled by pH: *in vivo* implications. J Biol Chem.

[20] Thompson D, Pepys MB, Wood SP (1999). The physiological structure of human C-reactive protein and its complex with phosphocholine. Structure.

[21] Xu C, Wang S, Thibault G, Ng DT (2013). Futile protein folding cycles in the ER are terminated by the unfolded protein O-mannosylation pathway. Science.

[22] Chang JY (2011). Diverse pathways of oxidative folding of disulfide proteins: underlying causes and folding models. Biochemistry.

[23] Maity H, Maity M, Krishna MM, Mayne L, Englander SW (2005). Protein folding: the stepwise assembly of foldon units. Proc Natl Acad Sci USA.

[24] Ji SR, Wu Y, Potempa LA, Qiu Q, Zhao J (2006). Interactions of C-reactive protein with low-density lipoproteins: implications for an active role of modified C-reactive protein in atherosclerosis. Int J Biochem Cell Biol.

[25] Ying SC, Gewurz H, Kinoshita CM, Potempa LA, Siegel JN (1989). Identification and partial characterization of multiple native and neoantigenic epitopes of human C-reactive protein by using monoclonal antibodies. J Immunol.

[26] Sockolosky JT, Szoka FC (2013). Periplasmic production via the pET expression system of soluble, bioactive human growth hormone. Protein Expr Purif.

[27] Ji SR (2007). Cell membranes and liposomes dissociate C-reactive protein (CRP) to form a new, biologically active structural intermediate: mCRP(m). FASEB J.

[28] Baltz ML (1982). Phylogenetic aspects of C-reactive protein and related proteins. Ann N Y Acad Sci.

[29] Guillon C (2014). A staggered decameric assembly of human C-reactive protein stabilized by zinc ions revealed by X-ray crystallography. Protein Pept Lett.

[30] Ramadan MA (2002). The three-dimensional structure of calcium-depleted human C-reactive protein from perfectly twinned crystals. Acta Crystallogr D Biol Crystallogr.

[31] Phillips JC (2005). Scalable molecular dynamics with NAMD. Journal of Computational Chemistry.

[32] MacKerell AD (1998). All-atom empirical potential for molecular modeling and dynamics studies of proteins. Journal of Physical Chemistry B.

[33] Humphrey W, Dalke A, Schulten K (1996). VMD: Visual molecular dynamics. Journal of Molecular Graphics.

