# Supplementary material for: Conformational folding and disulfide bonding drive distinct stages of protein structure formation
Source: Sci Rep. 2018 Jan 24;8:1494. doi: 10.1038/s41598-018-20014-y (PMC5784126; doi:10.1038/s41598-018-20014-y)
Supplement: Supplementary file 1 — Supplemental data [file 41598_2018_20014_MOESM1_ESM.pdf]

Conformational folding and disulfide bonding drive distinct stages of protein structure formation

Jian-Min Lv [1], Shou-Qin Lü [2], Juan Zhang [1], Zu-Pei Liu [1], Bo-Xuan Gao [1], Zhen-Yu Yao [1], Yue-Xin Wu [1], Lawrence A. Potempa [3], Shang-Rong Ji [1, *], Mian Long [2, *], Yi Wu [4, 5, *]

[1] MOE Key Laboratory of Cell Activities and Stress Adaptations, School of Life Sciences, Lanzhou University, Lanzhou 730000, P.R. China; [2] Center for Biomechanics and Bioengineering, Key Laboratory of Microgravity (National Microgravity Laboratory), and Beijing Key Laboratory of Engineered Construction and Mechanobiology, Institute of Mechanics, Chinese Academy of Sciences, Beijing 100190, P.R. China; [3] Roosevelt University College of Pharmacy, Schaumburg, IL 60173; [4] MOE Key Laboratory of Environment and Genes Related to Diseases, School of Basic Medical Sciences, Xi'an Jiaotong University, Xi'an, Shaanxi 710061, P.R. China; [5] Key Laboratory of Preclinical Study for New Drugs of Gansu Province, Lanzhou University, Lanzhou 730000, P.R. China.

[*] Corresponding authors: Dr. Yi Wu (Tel/Fax: 86-029-82657013; Email: wuy@lzu.edu.cn); Dr. Mian Long (Tel/Fax: 86-010-82544131; Email: mlong@imech.ac.cn); Dr. Shang-Rong Ji (Tel/Fax: 86-931-8914176; Email: jsr@lzu.edu.cn).

Table S1. Mutants for disulfide scanning of strand C to H of CRP subunit.

	Mutant	Disulfide bond	Strand pair
	CRP wt	C36-C97	C-H
Strands C to H	CRP C36S/H38C/H95C/C97S	C38-C95	C-H
	CRP C36S/S53C/L64C/C97S	C53-C64	D-E
	CRP C36S/I63C/V77C/C97S	C63-C77	E-F
	CRP C36S/T76C/E81C/C97S	C76-C81	F-G
Distant elements	CRP C36S/C97S/E108C/M161C	C108-C161	I-K
	CRP C36S/V86C/C97S/S149C	C86-C149	G*-J*

*Denotes the mutated residues that are located close to but not in the indicated strands.

Table S2. Mutants for disulfide scanning of strand I to M of CRP subunit.

	Mutant	Disulfide bond	Strand pair
	CRP wt	C36-C97	C-H
Versus strands C to H	CRP C36S/W110C	C97-C110	H-I
	CRP C36S/C97S/S99C/E108C	C99-C108	H-I
	CRP C36S/Y49C/C97S/Q150C	C49-C150	F-G*
	CRP C36S/T56C/C97S/A131C	C56-C131	D*-J*
	CRP C36S/L37C/C97S/V159C	C37-C159	C-K*
	CRP C36S/T41C/C97S/G154C	C41-C154	C*-K
	CRP A8C/C36S/H38C/C97S	C8-C38	A-C
	CRP S21C/C36S/S53C/C97S	C21-C53	B-D
Versus strands A & B	CRP A8C/C36S/C97S/G157C	C8-C157	A-K
	CRP V10C/C36S/C97S/D155C	C10-C155	A-K
	CRP S15C/C36S/C97S/G148C	C15-C148	B*-J*
	CRP S21C/C36S/C97S/I134C	C21-C134	B-J
	CRP K23C/C36S/C97S/S132C	C23-C132	B*-J
	CRP F9C/C36S/C97S/T200C	C9-C200	A-M
	CRP S18C/36S/97S/G196C	C18-C196	B*-L*
	CRP S21C/36S/97S/E193C	C21-C193	B-L

*Denotes the mutated residues that are located close to but not in the indicated strands.

Table S3. Summary of simulation set-up

System*	PDB	Features†	Simulations (ns)					
			Free MD	Steered MD‡				
				SA	SB	SC	SD	SE
Penta	1LJ7	$(C_a^{2+})^-/(S\text{-}S)^+$	100	5	5	4	8	4
	3PVO	$(C_a^{2+})^-/(S\text{-}S)^+$	100	6	4	4	4	4
	3PVO	$(C_a^{2+})^+/(S\text{-}S)^+$	100	5	4	3	4	4
Mono	3PVO	$(C_a^{2+})^-/(S\text{-}S)^-$	100	28	N/D	N/D	N/D	N/D
	3PVO	$(C_a^{2+})^-/(S\text{-}S)^+$	100	32	N/D	N/D	N/D	N/D

*Denotes Penta for pentamer and Mono for monomer. †defines $(C_a^{2+})^-/(S\text{-}S)^+$, $(C_a^{2+})^-/(S\text{-}S)^-$, $(C_a^{2+})^+/(S\text{-}S)^+$ as the simulation systems without calcium ion but with disulfide bond, without either calcium ion or disulfide bond, and with both calcium ions and disulfide bond, respectively. ‡Denotes SA/SB/SC/SD/SE as the respective subunit A/B/C/D/E.

Supplemental Figure Legends

Figure S1. Structural evolutions of CRP pentamers upon Steered Molecular Dynamics (SMD) simulations. Typical SMD processes with pulled subunit A were presented for the systems of 1LJ7(C_a^{2+})$^-$ (A, B, C), 3PVO(C_a^{2+})$^-$ (D, E, F), and 3PVO(C_a^{2+})$^+$ (G, H, I). Final snapshots of pentamer (*Cyan*) were shown in the left column (A, D, G) with the superimposition of initial conformation (*Orange*) upon the backbone alignment of other four fixed subunits. Corresponding snapshots of the pulled subunit alone were shown in the middle column (B, E, H) with the superimposition of its initial conformation upon backbone alignment. Quantitative relative (*Black*) and absolute (*Red*) RMSDs profiles were shown in the right column (C, F, I). The means ± SE of relative and absolute RMSDs at 3 ns for each subunit pulling simulations were presented in (J).

Figure S2. Subunit folding precedes pentamer assembly. (A) K13 and S120 locate at the assembly interface between two adjacent subunits in the crystal structure of CRP (PDB 1B09). They were mutated to cysteines to introduce inter-subunit disulfide bonds that are compatible with the pentameric structure. (B) Efficient secretion of CRP K13C/S120C mutant by COS-7 cells was detected by immunoblotting with CRP-8 mAb. The formation of the inter-subunit disulfide bonds in CRP K13C/S120C were revealed by the presence of reduction-sensitive oligomer band.

Figure S3. Strands C to H are folded to a near native conformation before

Cys36-Cys97 bonding. Secretory CRP mutants were expressed in *E. coli* cells. Each of these mutants harbors an inter-strand disulfide bond and has both Cys36 and Cys97 mutated to serine residues. Periplasm (top) and conditioned media (bottom) were examined by immunoblotting with 3H12 mAb. The ratio of correct inter-strand disulfide bonding was calculated as (band intensity of oxidized subunit) / (band intensity of oxidized subunit + band intensity of reduced subunit + band intensity of aggregates) using lanes without DTT treatment. The aggregates were composed of oligomers with inter-subunit disulfide crosslinking, as evidenced by their sensitivity to DTT treatment, and assemblies formed by reduced subunits, as evidenced by the gel profile of C36S/C97S mutant. * indicates the band of unprocessed CRP with signal peptide still attached as characterized by mass spectrometry.

Figure S4. The cytoplasmic stability of CRP subunit depends on N-terminal motifs. (A) Amino acid sequence of CRP subunit. Sequences corresponding to β-strand (blue) and α-helix (orange red) as described in the crystal structure of CRP (PDB 1B09) are indicated. (B) SDS-PAGE and Immunoblotting analysis. Lysates of *E. coli* cells with overexpressed CRP mutants were analyzed by 12 % SDS-PAGE and immunoblotting with 3H12 mAb or anti-strep antibodies. (C) Quantification of protein expression. Expression levels of mutant proteins were reduced by N-terminal truncations (left panel) but were not affected by C-terminal truncations (right panel). Each data point represents the mean of data obtained from at least 3 independent experiments and are presented as mean +/- S.E.

Figure S5. The intra-subunit disulfide bonding requires a.a. 168-176 helix.

Inclusion bodies formed by CRP mutants in *E. coli* cytoplasm were separated and solubilized by 8M urea. After passing through a desalting column to remove urea, the mutants were incubated in 20 mM Tris, 140 mM NaCl, PH 7.4 at 4 °C overnight. Samples with or without DTT reduction were analyzed by non-reducing SDS-PAGE and immunoblotting with 3H12 mAb. Oxidized subunits migrated faster than reduced counterparts (1,2). Quantification of the ratio of subunit oxidation is shown. The intra-subunit disulfide bond was found in the truncated CRP mutant Δ180-206, but not in the truncated CRP mutant Δ165-206, suggesting that a.a. 165-179 are required for the formation of the intra-subunit disulfide bond. Each data point represents the mean of data obtained from at least 3 independent experiments and are presented as mean +/- S.E.

Figure S6. Strands J and K are mislocated before Cys36-Cys97 bonding. Secretory CRP mutants were expressed in *E. coli* cells. Each of these mutants were constructed to include a unique inter-strand disulfide bond and to exclude the normal Cys36 and Cys97 bond by mutating these residues to serines. Conditioned media were examined by immunoblotting with 3H12 mAb. The ratio of correct inter-strand disulfide bonding was calculated as described in the legend to Figure S3. * Indicates the band of unprocessed CRP with signal peptide still attached as characterized by mass spectrometry.

Figure S7. Forced unfolding of monomer systems. Schematic force setting and extension-time profiles are presented in (A) and (B), respectively. Typical snapshots of the forced processes are shown in (C-F) for $(S\text{-}S)^-$ system and in (C'-F') for $(S\text{-}S)^+$

system. (S-S)$^-$ and (S-S)$^+$ denote the disulfide bond being disrupted and remaining intact, respectively. The snapshots are presented with Ser167-Gly177 α-helix in *Purple*, β-strands J and K (Ala131-Leu166) in *Green*, β-strands A, B (Gln1-Leu22) and L, M (Tyr192-Pro206) in *Blue* and others in *Orange*. The two amino acids involved in disulfide bond formation were shown in *Name Licorice* for clarity.

Figure S8. Evolutions of Hbond numbers between key adjacent strands during the forced unfolding simulations of monomer systems. Hbond numbers between the strands of C and H, H and I (A, D), the strands of D and E, E and F, F and G (B, E), and the strands of C and K, J and D (C, F) are presented for the monomer systems of (S-S)$^-$ (A, B, C) and (S-S)$^+$ (D, E, F), respectively. Data is shown as the slip average of adjacent twenty frames. The *dash vertical lines* depicted the moment that the interaction between strands of C and H was disrupted.

Figure S9. Comparisons of Hbond interactions between key secondary structures of monomer equilibration simulations. Data is presented as the mean ±SE of the last 50-ns trajectories. Here the capital letters represented the corresponding strand, helix-expressed Ser167-Gly177 helix, and loop-expressed Trp110-Pro115 loop.

Figure S10. Calcium binding defects do not affect the intra-subunit disulfide bonding. Secretory CRP mutants mutated at residues involved in CRP subunit calcium binding, were expressed in *E. coli* cells. Periplasm (top) and conditioned media (bottom) were examined by immunoblotting with 3H12 mAb. The ratio of

correct inter-strand disulfide bonding was calculated as described in the legend to Figure S3. * Indicates the band of unprocessed CRP with signal peptide still attached as characterized by mass spectrometry.

Figure S1

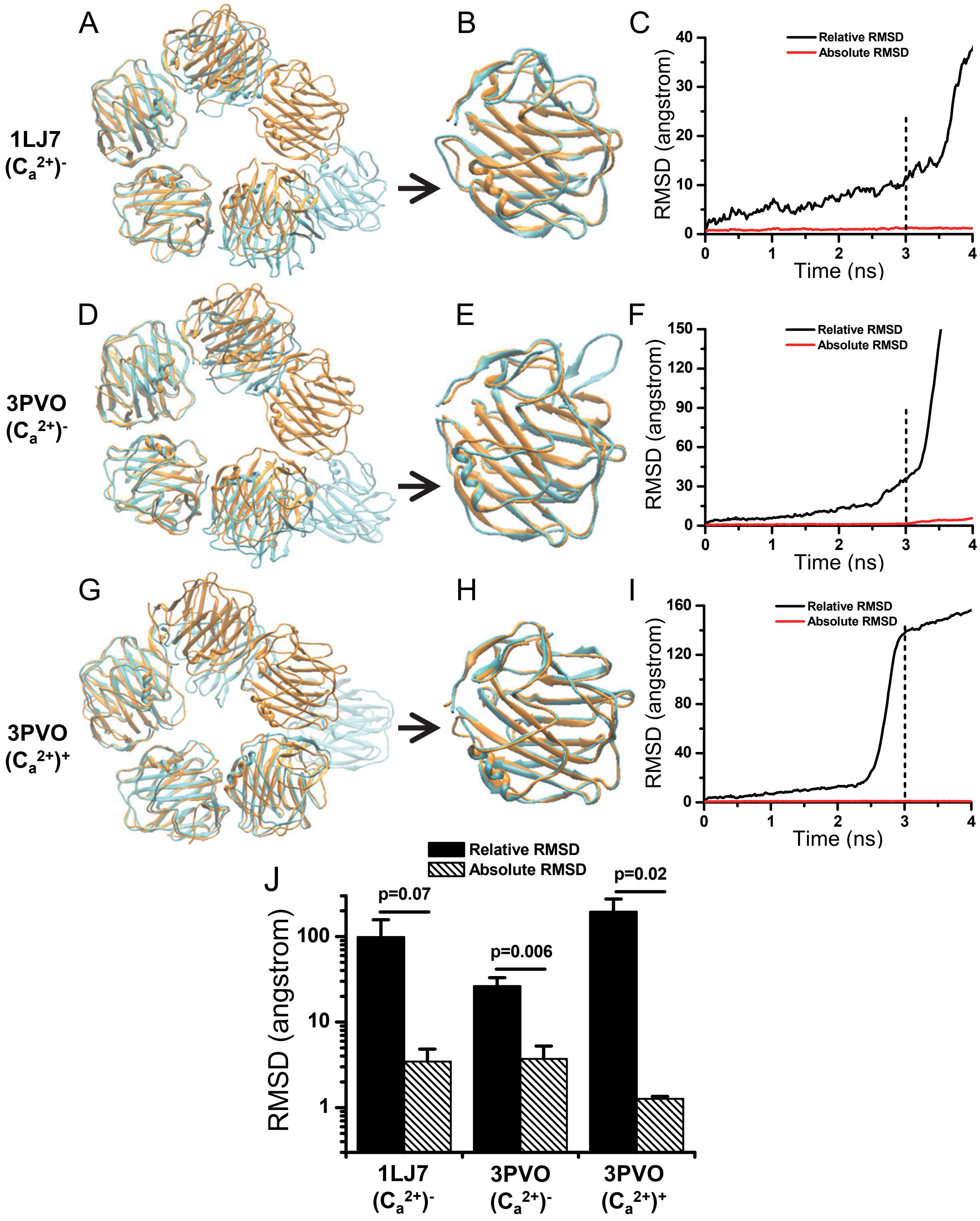

A
B
C
1LJ7
(Ca2+)-
Relative RMSD
Absolute RMSD
RMSD (angstrom)
Time (ns)
D
E
F
3PVO
(Ca2+)-
G
H
I
3PVO
(Ca2+)+
J
p=0.07
p=0.006
p=0.02
1LJ7 (Ca2+)-
3PVO (Ca2+)-
3PVO (Ca2+)+

Figure S2

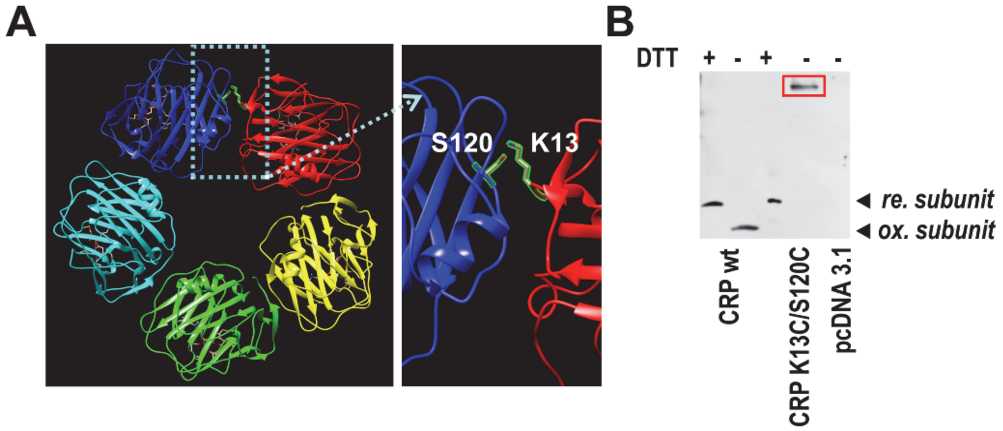
A
S120
K13
B
DTT
+
-
+
-
-
re. subunit
ox. subunit
CRP wt
CRP K13C/S120C
pcDNA 3.1

Figure S3

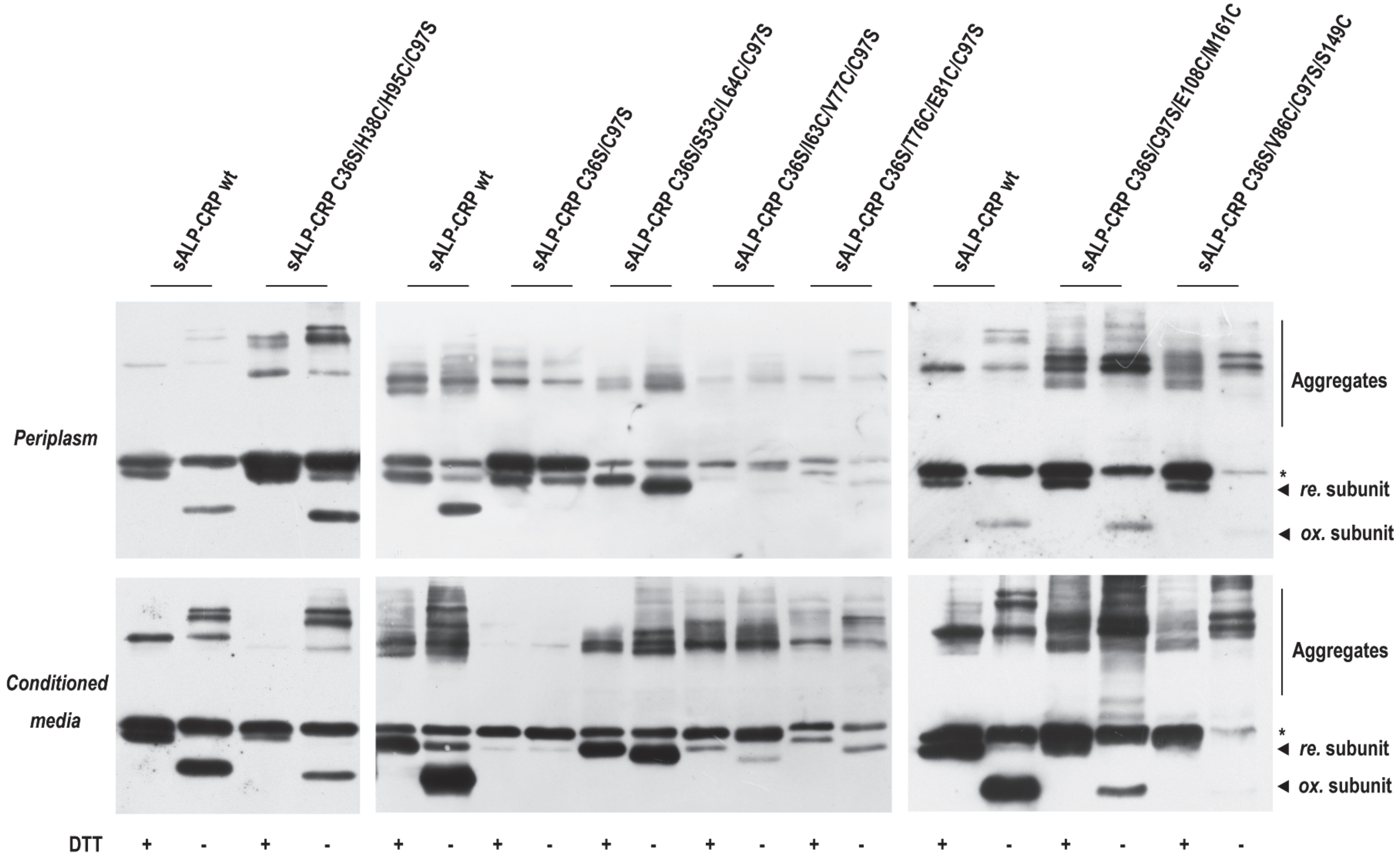
sALP-CRP wt
sALP-CRP C36S/H38C/H95C/C97S
sALP-CRP wt
sALP-CRP C36S/C97S
sALP-CRP C36S/S53C/L64C/C97S
sALP-CRP C36S/I63C/V77C/C97S
sALP-CRP C36S/T76C/E81C/C97S
sALP-CRP wt
sALP-CRP C36S/C97S/E108C/M161C
sALP-CRP C36S/V86C/C97S/S149C
Periplasm
Conditioned media
Aggregates
*
re. subunit
ox. subunit
Aggregates
*
re. subunit
ox. subunit
DTT
+ - + - + - + - + - + - + - + - + - + -

Figure S4

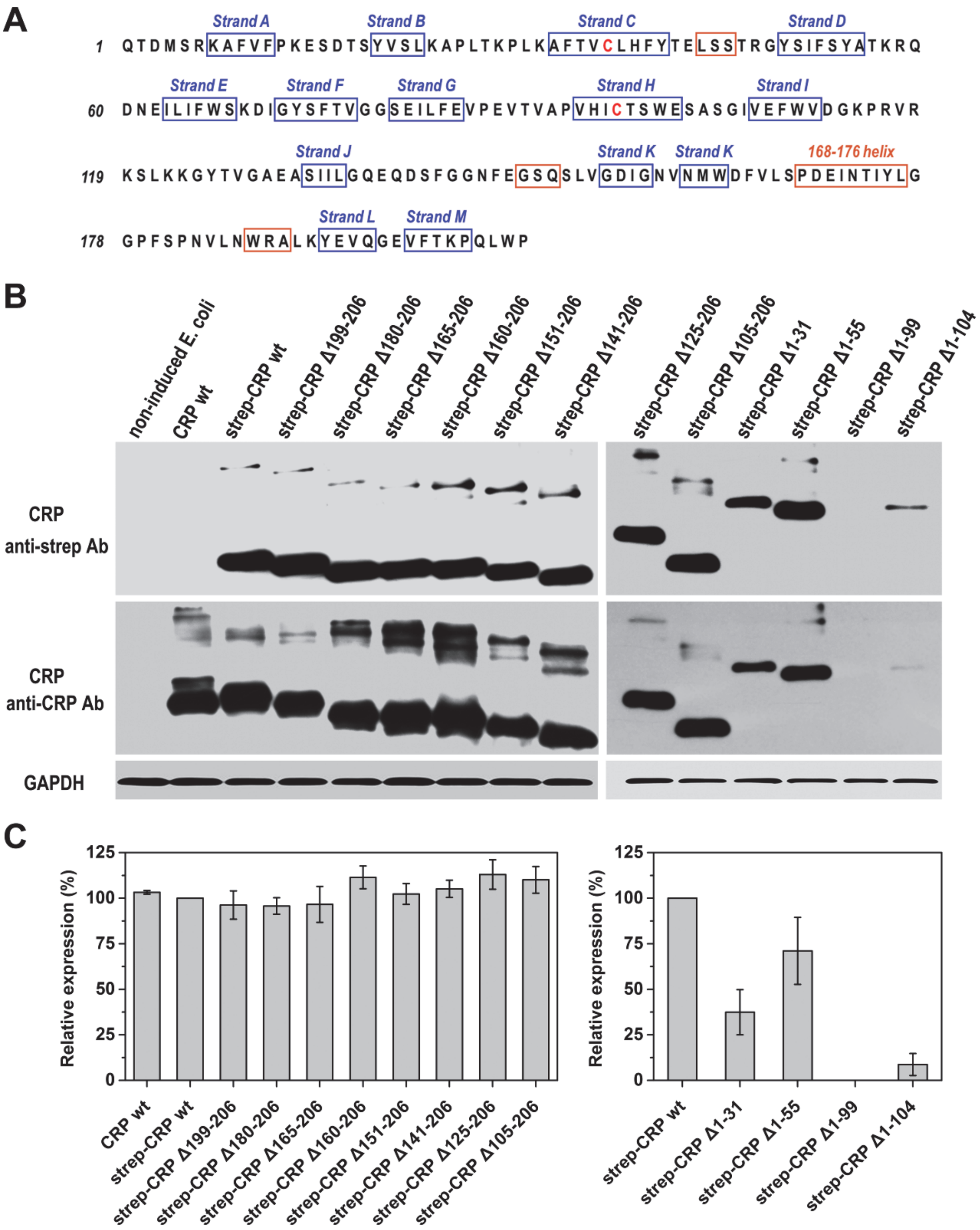
A
Strand A
Strand B
Strand C
Strand D
1 QTDMSRKAFVFPKESDTSYVSLKAPLTKPLKAFTVCLHFYTELSSTRGYSIFSYATKRQ
Strand E
Strand F
Strand G
Strand H
Strand I
60 DNEILIFWSKDIGYSFTVGGSEILFEVPEVTVAPVHICTSWESASGIVEFWVDGKPRVR
Strand J
Strand K
Strand K
168-176 helix
119 KSLKKGYTVGAEASIILGQEQDSFGGNFEGSQSLVGDIGNVNMWDFVLSPDEINTIYLG
Strand L
Strand M
178 GPFSPNVLNWRALKYEVQGEVFTKPQLWP
B
non-induced E. coli
CRP wt
strep-CRP wt
strep-CRP Δ199-206
strep-CRP Δ180-206
strep-CRP Δ165-206
strep-CRP Δ160-206
strep-CRP Δ151-206
strep-CRP Δ141-206
strep-CRP Δ125-206
strep-CRP Δ105-206
strep-CRP Δ1-31
strep-CRP Δ1-55
strep-CRP Δ1-99
strep-CRP Δ1-104
CRP
anti-strep Ab
CRP
anti-CRP Ab
GAPDH
C
Relative expression (%)
0
25
50
75
100
125
CRP wt
strep-CRP wt
strep-CRP Δ199-206
strep-CRP Δ180-206
strep-CRP Δ165-206
strep-CRP Δ160-206
strep-CRP Δ151-206
strep-CRP Δ141-206
strep-CRP Δ125-206
strep-CRP Δ105-206
Relative expression (%)
0
25
50
75
100
125
strep-CRP wt
strep-CRP Δ1-31
strep-CRP Δ1-55
strep-CRP Δ1-99
strep-CRP Δ1-104

Figure S5

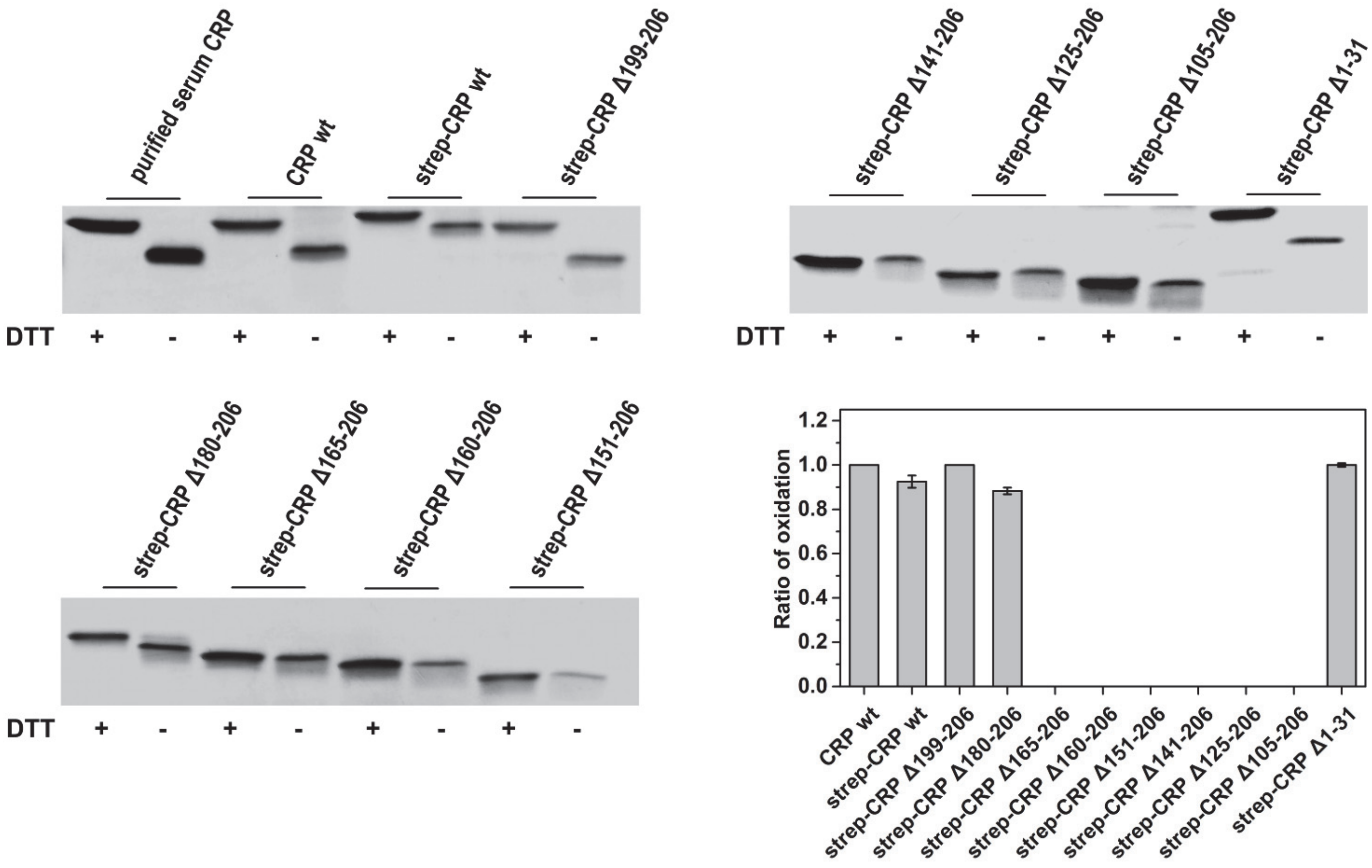
purified serum CRP
CRP wt
strep-CRP wt
strep-CRP Δ199-206
DTT + - + - + - + -
strep-CRP Δ141-206
strep-CRP Δ125-206
strep-CRP Δ105-206
strep-CRP Δ1-31
DTT + - + - + - + -
strep-CRP Δ180-206
strep-CRP Δ165-206
strep-CRP Δ160-206
strep-CRP Δ151-206
DTT + - + - + - + -
Ratio of oxidation
1.2
1.0
0.8
0.6
0.4
0.2
0.0
CRP wt
strep-CRP wt
strep-CRP Δ199-206
strep-CRP Δ180-206
strep-CRP Δ165-206
strep-CRP Δ160-206
strep-CRP Δ151-206
strep-CRP Δ141-206
strep-CRP Δ125-206
strep-CRP Δ105-206
strep-CRP Δ1-31

Figure S6

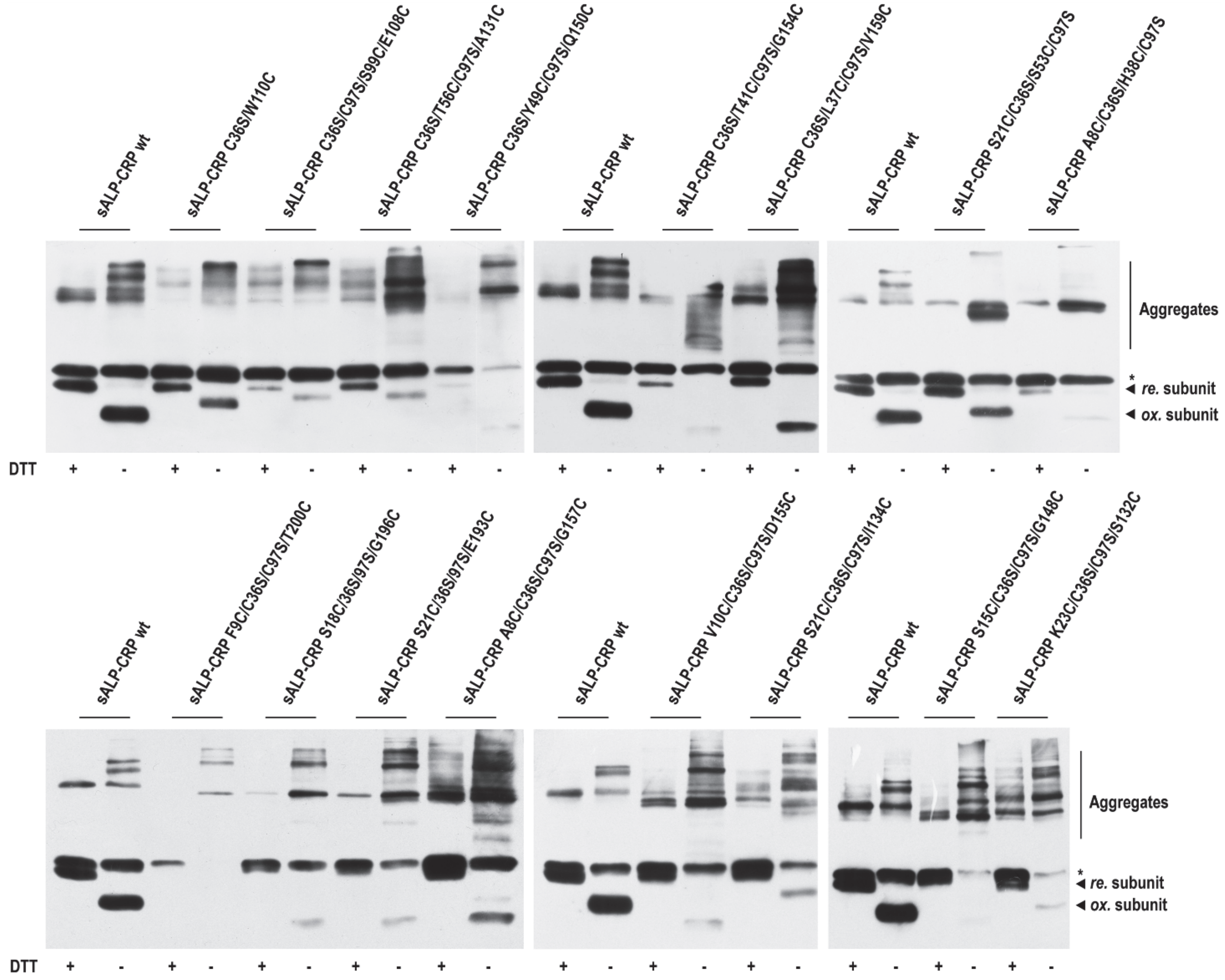
sALP-CRP wt
sALP-CRP C36S/W110C
sALP-CRP C36S/C97S/S99C/E108C
sALP-CRP C36S/T56C/C97S/A131C
sALP-CRP C36S/Y49C/C97S/Q150C
sALP-CRP wt
sALP-CRP C36S/T41C/C97S/G154C
sALP-CRP C36S/L37C/C97S/V159C
sALP-CRP wt
sALP-CRP S21C/C36S/S53C/C97S
sALP-CRP A8C/C36S/H38C/C97S
Aggregates
*
re. subunit
ox. subunit
DTT
+ - + - + - + - + - + - + - + - + - + - + -
sALP-CRP wt
sALP-CRP F9C/C36S/C97S/T200C
sALP-CRP S18C/36S/97S/G196C
sALP-CRP S21C/36S/97S/E193C
sALP-CRP A8C/C36S/C97S/G157C
sALP-CRP wt
sALP-CRP V10C/C36S/C97S/D155C
sALP-CRP S21C/C36S/C97S/I134C
sALP-CRP wt
sALP-CRP S15C/C36S/C97S/G148C
sALP-CRP K23C/C36S/C97S/S132C
Aggregates
*
re. subunit
ox. subunit
DTT
+ - + - + - + - + - + - + - + - + - + - + -

Figure S7

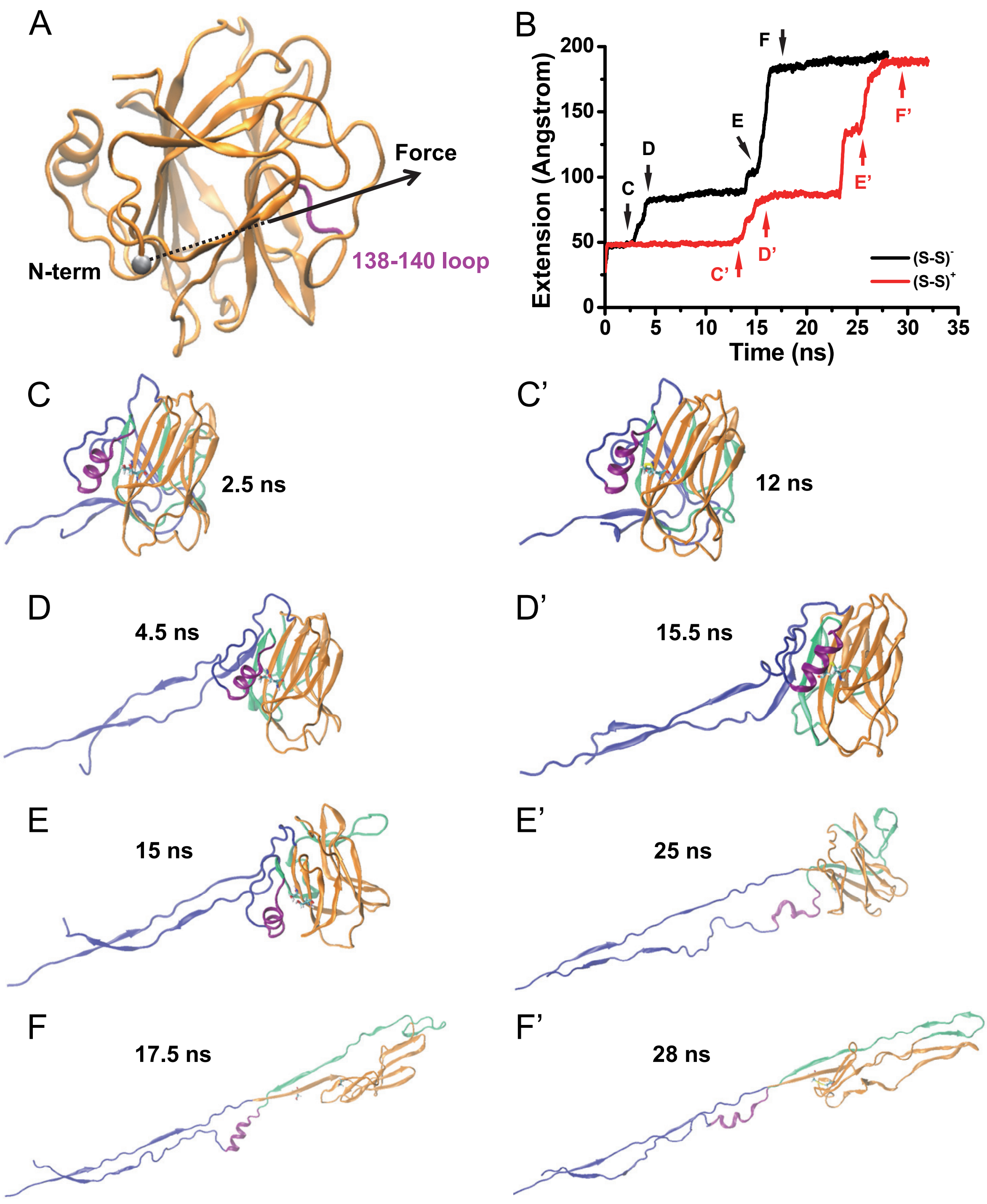
A
Force
N-term
138-140 loop
B
F
E
D
C
F'
E'
D'
C'
Extension (Angstrom)
Time (ns)
(S-S)⁻
(S-S)⁺
C
2.5 ns
C'
12 ns
D
4.5 ns
D'
15.5 ns
E
15 ns
E'
25 ns
F
17.5 ns
F'
28 ns

Figure S8

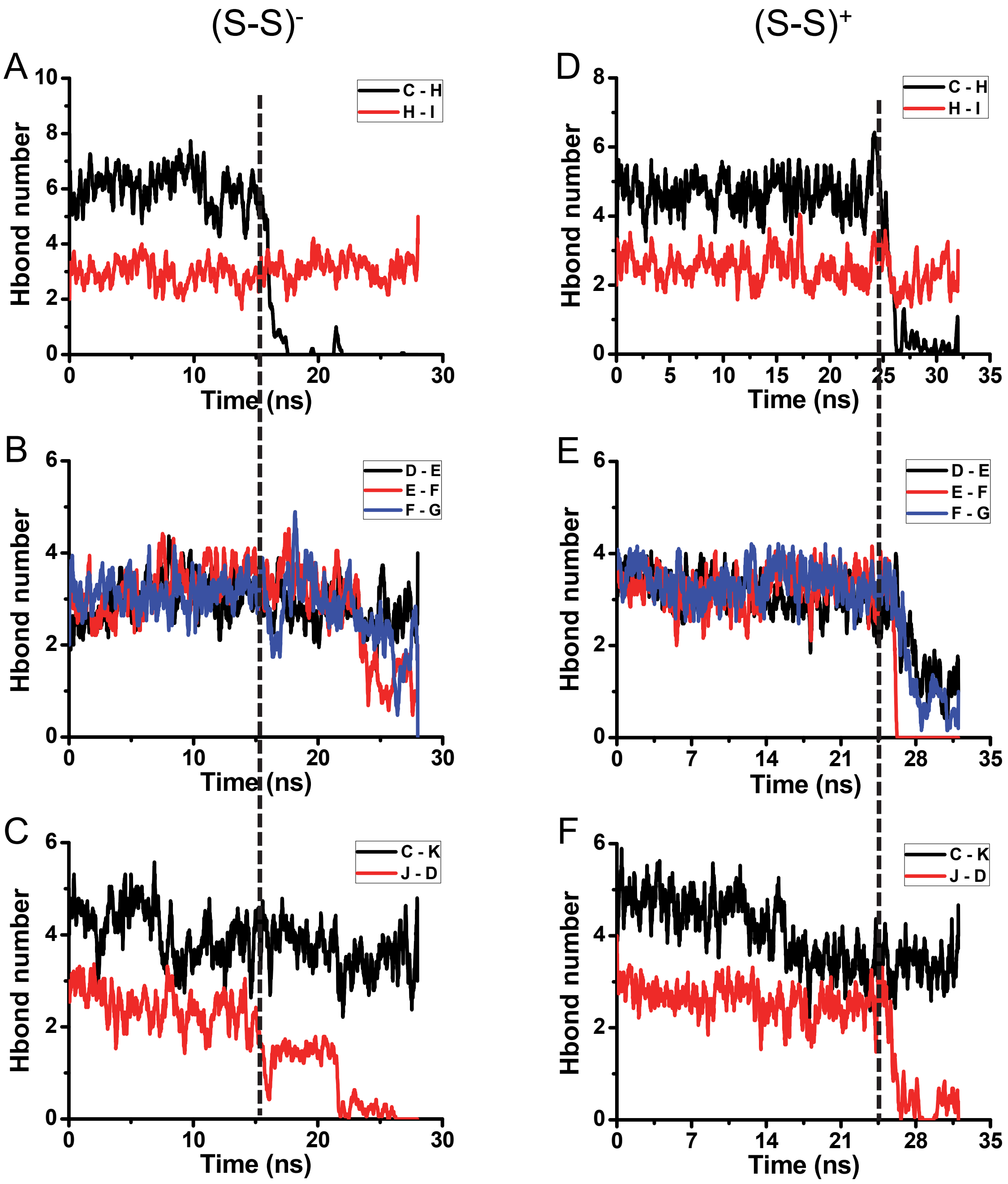

Figure S9

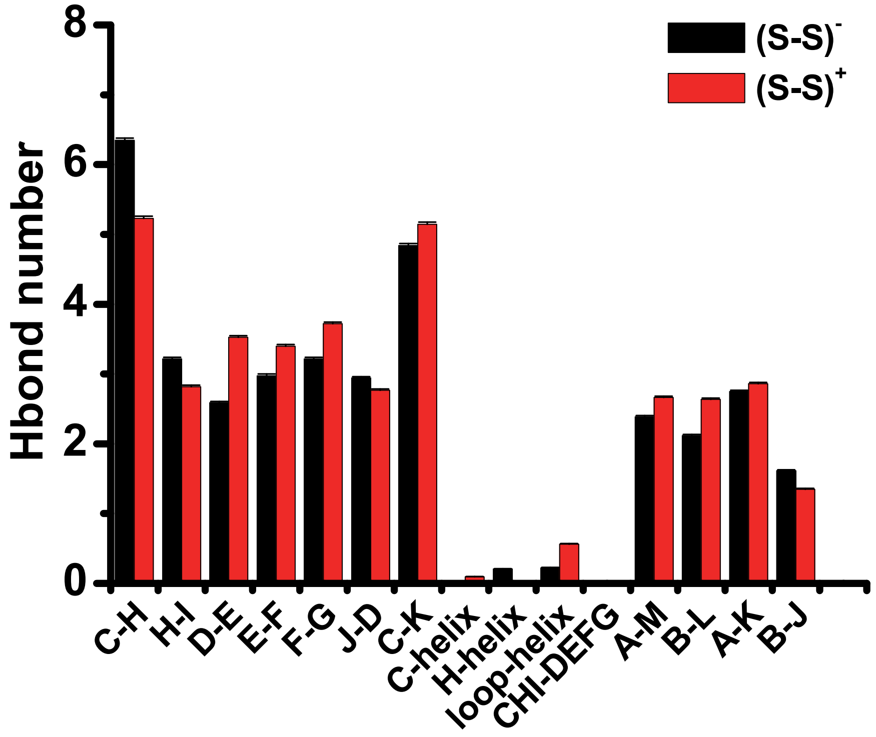

(S-S)-
(S-S)+
Hbond number
0
2
4
6
8
C-H
H-I
D-E
E-F
F-G
J-D
C-K
C-helix
H-helix
loop-helix
CHI-DEFG
A-M
B-L
A-K
B-J

Figure S10

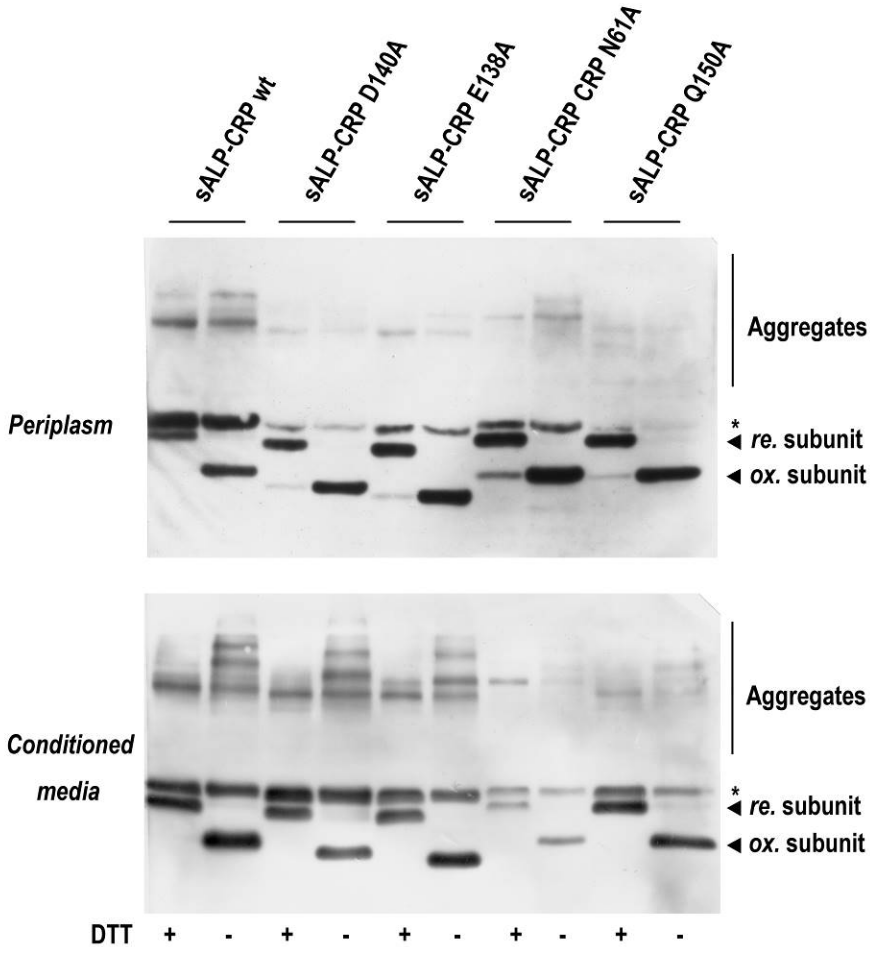
sALP-CRP wt
sALP-CRP D140A
sALP-CRP E138A
sALP-CRP CRP N61A
sALP-CRP Q150A
Periplasm
Aggregates
*
◄ re. subunit
◄ ox. subunit
Conditioned media
Aggregates
*
◄ re. subunit
◄ ox. subunit
DTT + - + - + - + - + -

References

1. Wang, M. Y., Ji, S. R., Bai, C. J., El Kebir, D., Li, H. Y., Shi, J. M., Zhu, W., Costantino, S., Zhou, H. H., Potempa, L. A., Zhao, J., Filep, J. G., and Wu, Y. (2011) A redox switch in C-reactive protein modulates activation of endothelial cells. *FASEB J* **25**, 3186-3196
2. Baltz, M. L., de Beer, F. C., Feinstein, A., Munn, E. A., Milstein, C. P., Fletcher, T. C., March, J. F., Taylor, J., Bruton, C., Clamp, J. R., Davies, A. J., and Pepys, M. B. (1982) Phylogenetic aspects of C-reactive protein and related proteins. *Ann N Y Acad Sci* **389**, 49-75
